# Case Report: IgG4-related disease presenting with prominent oculomotor nerve palsy

**DOI:** 10.3389/fneur.2026.1829911

**Published:** 2026-07-09

**Authors:** Lishi Yu, Weidong Huang, Yifei Xu, Chunhong Zhang, Honghua Lv, Wenhui Lei

**Affiliations:** 1 Department of Rheumatology, The Fifth Affiliated Hospital of Wenzhou Medical University, Lishui, Zhejiang, China; 2 Department of Nephrology, The Fifth Affiliated Hospital of Wenzhou Medical University, Lishui, Zhejiang, China

**Keywords:** case report, cranial neuropathy, IgG4-related disease, oculomotor nerve palsy, treatment

## Abstract

**Background:**

Immunoglobulin G4-related disease (IgG4-RD) is a systemic fibroinflammatory disorder that uncommonly involves cranial nerves. Oculomotor nerve palsy as the predominant manifestation of IgG4-RD is exceptionally rare and may elude timely diagnosis due to nonspecific symptoms.

**Case presentation:**

A 76-year-old man presented with a two-month history of bilateral plantar numbness and pain, followed by nausea, vomiting, and headache. The patient was hospitalized twice: first in August 2025 in the Department of Hematology, and subsequently in October 2025 in the Department of Rheumatology and Immunology. Laboratory evaluation revealed markedly elevated immunoglobulins, prompting a comprehensive workup including bone marrow aspiration, positron emission tomography-computed tomography (PET-CT), and lymph node biopsy. Histopathology demonstrated reactive lymphoid hyperplasia with abundant IgG4 + plasma cell infiltration (IgG4+ > 100 cells/HPF; IgG4/IgG ratio >40%), which established the diagnosis of IgG4-RD according to the 2019 ACR/EULAR classification criteria. During the second hospitalization, the patient developed right ptosis and diplopia, and neurological examination confirmed isolated right oculomotor nerve palsy. Contrast-enhanced brain MRI showed no structural abnormalities, and CTA, along with CSF analysis, excluded alternative etiologies such as aneurysm, neoplasm, or infection. Treatment with intravenous methylprednisolone (40 mg daily) followed by oral mycophenolate mofetil (500 mg twice daily) led to significant clinical improvement of oculomotor palsy within 4 months, with normalization of serum IgG4 levels (from a peak of 74.3 g/L to 8.94 g/L at follow-up) and reduction of inflammatory markers.

**Conclusion:**

IgG4-RD should be considered in the differential diagnosis of unexplained cranial neuropathies, even in the absence of radiographic abnormalities. This case highlights that neurological recovery may be gradual and require sustained immunosuppression, and underscores the importance of multidisciplinary collaboration in managing atypical manifestations of IgG4-RD.

## Introduction

Immunoglobulin G4–related disease (IgG4-RD) is a systemic immune-mediated condition characterized by infiltration of IgG4 + plasma cells, tissue fibrosis, and multiorgan involvement, presenting with a broad spectrum of clinical phenotypes. The pancreas, lacrimal glands, salivary glands, and kidneys are among the most commonly affected organs (1, 2). The orbit is a recognized predilection site for IgG4-RD, often presenting with lacrimal gland enlargement and extraocular muscle involvement; however, cranial nerve involvement, particularly of the oculomotor nerve, is exceptionally rare. Pooled analyses of prior studies suggest an incidence of less than 1% among all IgG4-RD patients (3, 4).

The oculomotor nerve arises from the midbrain and traverses the cavernous sinus and superior orbital fissure to reach the orbit, lying in close anatomical proximity to the orbital apex and cavernous sinus—regions frequently targeted by IgG4-RD. Pathophysiological mechanisms relevant to nerve palsy in this context include inflammation-mediated compression, immune complex deposition, and direct neural infiltration (4, 5).

Recent refinements in diagnostic criteria for IgG4-RD and advances in imaging modalities have progressively improved the detection of ophthalmic involvement. Nonetheless, oculomotor nerve palsy remains an atypical manifestation that often eludes timely diagnosis due to nonspecific symptoms and may be misattributed to vascular lesions, intracranial neoplasms, or other autoimmune disorders (6). Moreover, IgG4-RD is recognized as a potential pre-lymphomatous condition, conferring a significantly elevated risk of lymphoma development during long-term follow-up compared with the general population (7, 8). The clinical picture can be further complicated by concomitant autoantibody positivity, including anti-neutrophil cytoplasmic antibodies (ANCA) (9–11).

In this report, we describe a case of IgG4-RD complicated by oculomotor nerve palsy and provide a systematic discussion of its pathogenesis, therapeutic management, and prognostic implications. The aim is to enhance clinical recognition of this rare presentation and to provide diagnostic and therapeutic insights for atypical manifestations of IgG4-RD.

## Case presentation

The patient, a 76-year-old man, was first admitted to the Department of Hematology from August 8 to August 15, 2025. He presented with bilateral plantar numbness and pain for more than two months, accompanied by nausea and vomiting for four days. The plantar symptoms began insidiously about two months prior, without associated chest discomfort, palpitations, headache, abdominal symptoms, or constitutional symptoms. After initial evaluation at a local hospital, analgesics provided temporary relief of the plantar symptoms.

Subsequently, the patient developed headaches and vomiting, prompting further evaluation at the same institution. Laboratory studies revealed markedly elevated immunoglobulins. During his first hospitalization (August 8–15, 2025), peripheral blood testing showed a substantial elevation in IgG levels. A comprehensive diagnostic workup, including bone marrow aspiration and biopsy, positron emission tomography–computed tomography (PET-CT), lymph node biopsy, and immunohistochemical staining, was performed. Based on these findings, a preliminary diagnosis of immunoglobulin G4–related disease (IgG4-RD) was considered. However, the patient perceived spontaneous symptomatic improvement and elected to discontinue further treatment before a definitive diagnosis or therapy could be instituted.

On October 9, 2025, the patient was readmitted to the Department of Rheumatology and Immunology due to recurrence of nausea and vomiting, which was this time accompanied by headache, ptosis of the right eyelid, diplopia, persistent bilateral lower-extremity numbness, poor appetite, and generalized fatigue. The patient’s medical history was notable for the absence of hypertension or diabetes mellitus, and he denied alcohol or tobacco use. On admission, the patient’s vital signs were temperature 37 °C, respiratory rate 20 breaths/min, heart rate 75 beats/min, and blood pressure 154/93 mmHg.

Neurological examination showed mild ptosis of the upper right eyelid, with the right eye in a “downward and outward” position, slightly dilated pupils, presence of light reflex, normal range of motion in the left eye, and no abnormalities observed in other cranial nerve examinations. Cardiopulmonary and abdominal examinations were unremarkable, with no peripheral edema.

**Figure 1:**
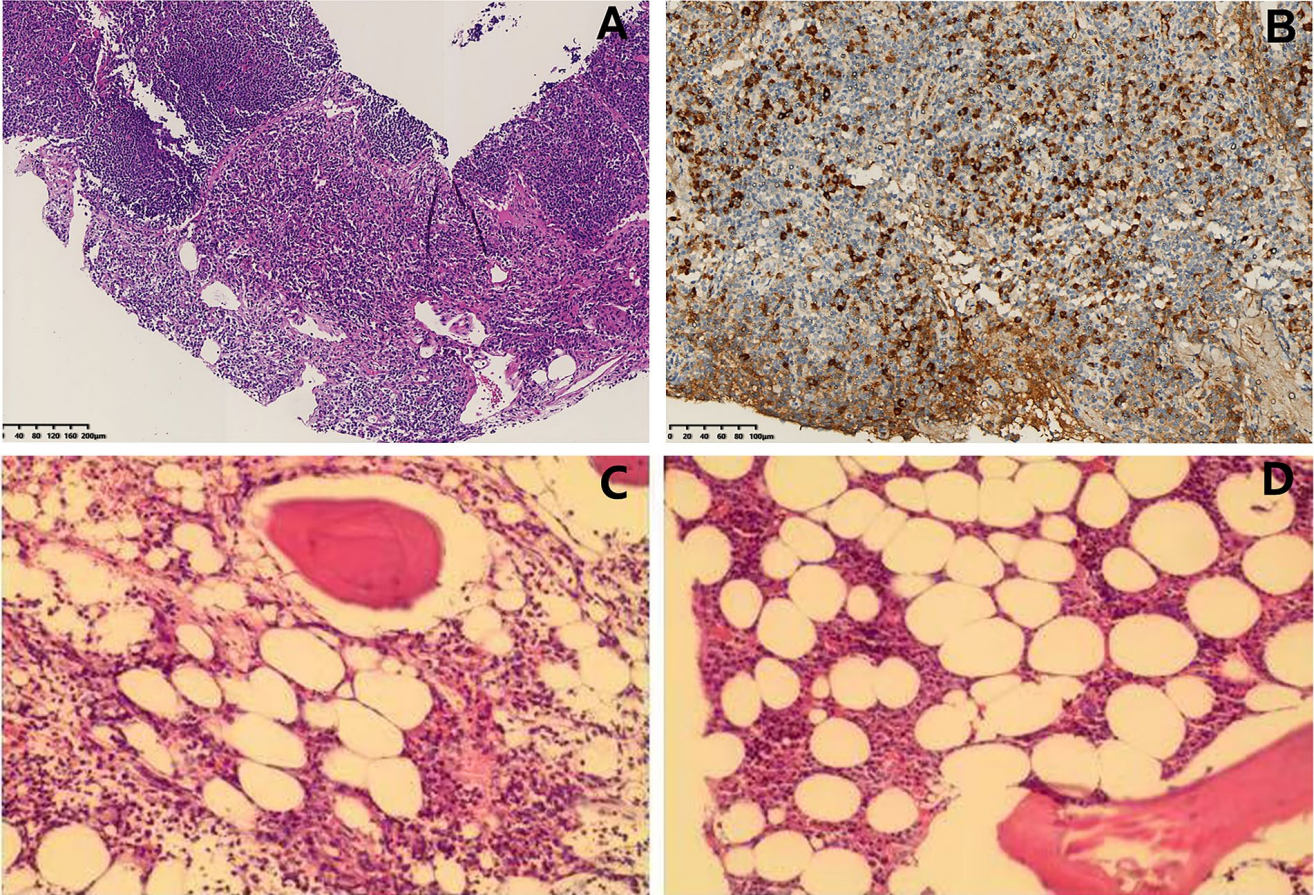
Pathological characteristics of lymph nodes and bone marrow tissue in patients. **(A)** Lymph node biopsy, hematoxylin and eosin (HE) staining, showed dense infiltration of lymphoplasmacytes (scale bar = 200 μm). **(B)** Lymph node biopsy immunohistochemistry showed a large number of IgG4-positive plasma cells with an IgG4/IgG ratio>40%. (Scale bar = 100 μm). **(C)** Bone marrow biopsy, HE staining, shows mixed hematopoietic and adipose tissue with scattered hematopoietic cells and vacuolated adipocytes. **(D)** Scattered hematopoietic cells with a prominent bony trabecula at the periphery.

Laboratory and imaging investigations revealed the following key findings from both the hematology and current rheumatology admissions, summarized in Table 1. Serum IgG4 level at initial presentation was 74.3 g/L (normal range 0.03–2.01 g/L). Anti-neutrophil cytoplasmic antibody (ANCA) testing was negative. PET-CT showed enlargement of lymph nodes throughout the body, increased glucose metabolism, and mild elevation of splenic parenchymal metabolism. These findings prompted consideration of hematologic diseases. (Supplementary Figure S1). Bone marrow biopsy from the right posterior superior iliac spine demonstrated increased yellow marrow content (hematopoietic to adipose ratio 30:70) with a granulocytic-to-erythroid ratio of 2–3:1, active granulopoiesis (predominantly metamyelocytes and later stages) and active erythropoiesis (intermediate to late normoblasts), with 4–6 megakaryocytes per high-power field and scattered mature plasma cells; reticulin staining was negative (Figure 1). Histopathological evaluation of the right axillary lymph node revealed reactive lymphoid hyperplasia with prominent interfollicular plasma cell proliferation; immunohistochemistry suggested possible IgG4-related lymphadenopathy, necessitating correlation with clinical, imaging, and laboratory data. Immunophenotyping demonstrated CD20 + B cells, CD3 + and PD-1 + T cells, CD21 + follicular dendritic networks, CD10 + germinal centers, and high Ki-67 in germinal centers, with scattered CD30 + cells; plasma cells expressed CD138, MUM1, IgG, and IgG4 (hot spots >100 cells per high-power field; IgG4/IgG ratio >40%), and there was kappa light chain restriction with partial lambda positivity (Figure 1), Additionally, *in situ* hybridization for Epstein–Barr virus-encoded small RNA (EBER) was negative, ruling out EBV-associated lymphoproliferative disorders.

**Table 1:** Laboratory findings during the patient’s two hospital admissions.

The laboratory parameters	Result 1^1^	Result 2^2^	Reference range
C-reactive protein (mg/L)	27.94	1.82	<8
Ferritin (ng/ml)	487.5	314.7	21.8–274.2
Immunoglobulin G (g/L)	48.93	59.24	7–16
Complement C3 (g/L)	0.83	0.87	0.9–1.8
Globulin (g/L)	78.8	71.4	20–40
IgG4(g/L)	63.8	74.3	0.03–2.01
Anti-nuclear antibody	Positive	/	Negative
Fluorescent pattern	Cytoplasmic	/	Negative
Anti-nuclear antibody titer	1:160	/	<1:80
24-hour urinary protein (g/24h)	0.56	/	<0.15
Anti-neutrophil cytoplasmic antibody	Negative	/	Negative

^1^Data from the patient's previous admission to the Department of Hematology.

^2^Data from the current admission to the Department of Rheumatology and Immunology.

The diagnostic criteria for IgG4-RD, according to the 2019 American College of Rheumatology/European League Against Rheumatism (ACR/EULAR) International Consensus Classification Criteria for IgG4-Related Disease, were unequivocally satisfied. Intravenous methylprednisolone at 40 mg daily (approximately 0.6 mg/kg/day) was started on day 1 of admission, a dose chosen to balance efficacy and safety given the patient’s advanced age and concerns about corticosteroid-related side effects such as hypertension, hyperglycemia, and osteoporosis. Initially, the patient’s right ptosis was mild and did not raise immediate concern; however, during glucocorticoid therapy, the ptosis paradoxically progressed, accompanied by ipsilateral forehead wrinkling (Supplementary Figure S2A). Neurological examination revealed complete ptosis of the right upper eyelid, with the right eye in a “down and out” (abducted and slightly depressed) position, dilated pupil, and loss of light reflex, consistent with right oculomotor nerve palsy. The left eye had a normal range of motion, and no abnormalities were found in other cranial nerve examinations.

To exclude alternative etiologies, additional investigations were pursued. Head and neck CTA revealed no evidence of aneurysms, vascular compression, or occlusive diseases. Contrast-enhanced brain MRI was performed on a 3.0 T scanner using thin-layer (1 mm) sequences targeting the orbital and cavernous sinus regions, including T1-weighted, T2-weighted, and post-contrast fat suppression sequences; no perineural thickening, enhancement, signal abnormality, or cavernous sinus asymmetry were identified (Supplementary Figure S1). Visual evoked potentials (VEP) and CSF analysis were also negative (Supplementary Figure S3; Supplementary Table 1). Regarding the differential diagnosis of oculomotor nerve palsy, microvascular ischemic neuropathy was considered but excluded for several reasons: (1) the patient lacked typical vascular risk factors (normotensive after initial elevation, non-diabetic); (2) the presentation did not show the typical pupil-sparing pattern of microvascular palsy; (3) the subacute progression over days rather than acute onset is atypical for microvascular ischemia; (4) significant improvement of oculomotor symptoms following glucocorticoid therapy provides counter-evidence against ischemic etiology as the primary cause; (5) normal brain MRI with no acute ischemic lesions on diffusion-weighted imaging further argues against microvascular infarction. Neoplastic and paraneoplastic processes were excluded through whole-body imaging and CSF analysis. Other inflammatory neuropathies (e.g., granulomatosis with polyangiitis, eosinophilic granulomatosis with polyangiitis) were ruled out, given negative ANCA testing and the absence of systemic symptoms such as uveitis or sinus involvement typically present in these diseases. Following the exclusion of other causes, glucocorticoid therapy was continued. According to the 2015 International Consensus Guidance Statement on the Management and Treatment of IgG4-Related Disease, and given the severity of the disease (oculomotor nerve involvement), a single dose of cyclophosphamide (0.4 g) was administered intravenously on the 5th day of hospitalization (12). However, due to the need for close monitoring of cyclophosphamide administration in the hospital, the patient found this treatment option inconvenient and requested a switch to oral medication. Considering the favorable safety profile of MMF in elderly patients, mycophenolate mofetil (MMF) 500 mg twice daily was prescribed as a steroid-sparing agent. Rituximab was also under consideration, but this treatment required the patient to pay a significant fee; therefore, the patient did not agree to its use. The patient was discharged on hospital day 6 with the oral regimen.

## Outcome and follow-up

**Figure 2:**
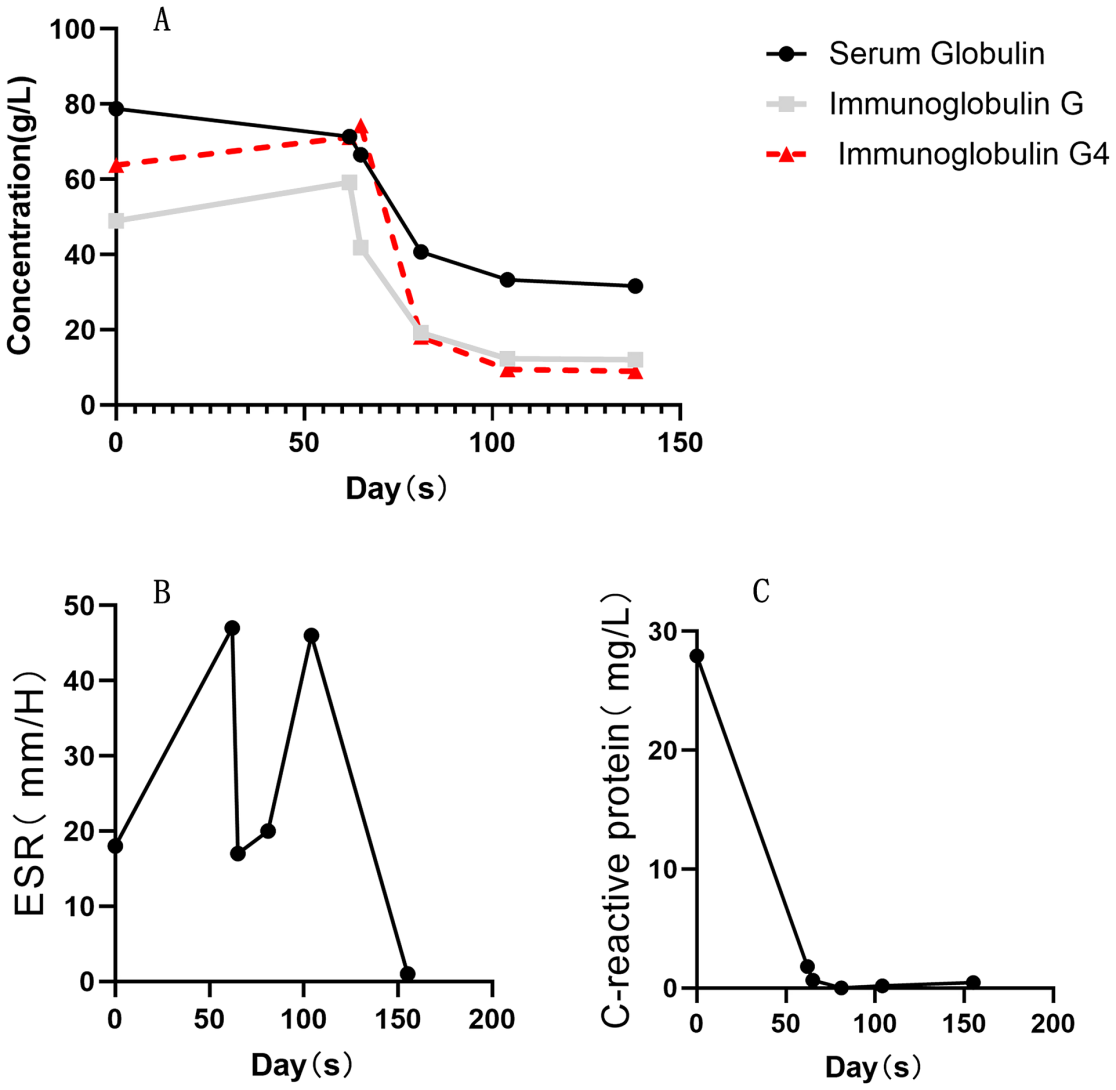
Serial changes in laboratory parameters during follow-up. **(A)** Serum globulin, IgG, and IgG4 levels (g/L). **(B)** Erythrocyte sedimentation rate (ESR) (mm/h). **(C)** C-reactive protein (CRP) level (mg/L). The *x*-axis represents time points (days).

**Figure 3:**
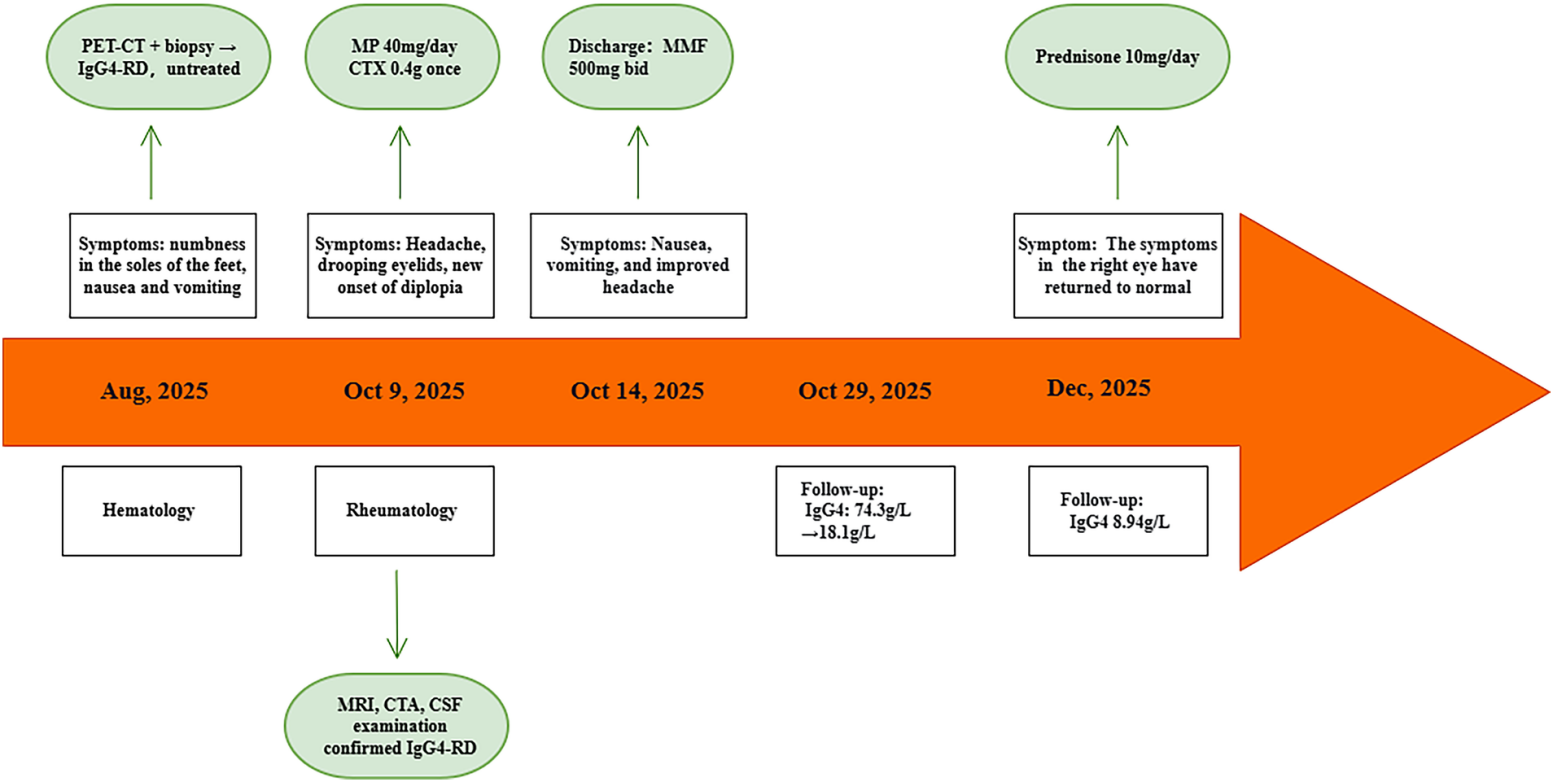
Clinical timeline of diagnosis, treatment, and follow-up. MP, methylprednisolone; CTX, cyclophosphamide; MMF, mycophenolate mofetil; IgG4-RD, immunoglobulin G4-related disease; PET-CT, positron emission tomography-computed tomography; CTA, computed tomography angiography; CSF, cerebrospinal fluid.

After four months of glucocorticoid and immunosuppressive therapy, the patient achieved significant clinical improvement with near-complete resolution of ptosis with restoration of full extraocular movements in all directions and preserved visual function, enabling unrestricted daily activities (Supplementary Figure S2B). Serum IgG4 levels decreased to 8.94 g/L (normal range 0.03–2.01 g/L), and the erythrocyte sedimentation rate, C-reactive protein, and globulin concentrations were reduced to standard levels (Figure 2). The glucocorticoid dose was tapered successfully to prednisone 10 mg daily. The patient reported excellent subjective well-being and returned to baseline functional status. A detailed timeline is provided in Figure 3.

Notable treatment-related adverse effects included weight gain and dyslipidemia, attributed to corticosteroid therapy. Ongoing longitudinal follow-up is planned to further minimize glucocorticoids and mitigate potential long-term medication-related complications.

## Discussion

Cranial nerve involvement in IgG4-related disease (IgG4-RD) is uncommon, with existing literature predominantly describing trigeminal (13–15), optic (16, 17), and facial nerve (18, 19) manifestations. Oculomotor nerve palsy remains particularly rare and is typically attributed to cavernous sinus involvement. Here, we reported a case of isolated oculomotor nerve palsy in IgG4-RD without identifiable abnormalities on contrast-enhanced brain MRI, and we discussed potential underlying mechanisms.

Recent structured radiological studies have increasingly recognized head and neck involvement patterns in IgG4-RD, including orbital, salivary gland, and skull base/cranial nerve manifestations. Pehlivan et al. (20) highlighted the role of MRI in detecting subtle neural involvement, emphasizing that dedicated thin-section sequences may reveal perineural enhancement or cavernous sinus asymmetry not apparent on standard imaging. We specifically evaluated our patient’s imaging for two key features, namely perineural spread and pachymeningeal enhancement, which are commonly highlighted in recent structured radiological studies of the skull base. Regarding perineural spread, thin-slice MRI was systematically reviewed for neural foramen enlargement or tubular enhancement along the trigeminal nerve branches and oculomotor nerve; none was identified. Regarding pachymeningeal enhancement, we evaluated the dura along the cerebral convexities, falx cerebri, tentorium cerebelli, and skull base; no abnormal dural thickening or enhancement was observed. The systematic exclusion of these features strengthens our diagnostic reasoning and points toward alternative mechanisms, such as anatomical vulnerability or immune-mediated neuritis below current imaging resolution. In our case, although subtle imaging findings such as perineural enhancement or cavernous sinus asymmetry were specifically assessed by neuroradiology specialists and found to be absent, the absence of detectable structural abnormalities does not exclude a pathophysiological role for anatomical vulnerability or low-grade, diffuse inflammation below current imaging resolution. To explore how such MRI-negative neural injury might occur, anatomical and mechanical factors warrant consideration. The oculomotor nerve traverses the superior orbital fissure into the orbit, lying in proximity (within ~2 mm) to extraocular muscles, lacrimal structures, and the cavernous sinus wall. The orbit’s inherently narrow apex constitutes a locus minoris resistentiae for inflammatory attack in IgG4-RD (21). In a Korean multicenter retrospective imaging–clinical cohort of 42 patients with IgG4-related ophthalmic disease, trigeminal branch thickening occurred in 11.9% of cases, and over half of these had concurrent oculomotor or abducens nerve palsy. Multivariable analysis showed that each 1 mm increase in orbital apex soft tissue thickness significantly raised the risk of nerve palsy (OR = 2.1, 95% CI 1.3–3.4), suggesting mechanical compression from soft tissue swelling as a key driver of oculomotor dysfunction (22). This anatomical mechanism should be considered alongside the clinical presentation, particularly the presence of pupil involvement. This anatomical vulnerability also provides a pathological basis for us to understand the oculomotor nerve paralysis present in this patient.

The presence of pupil involvement in oculomotor nerve palsy typically raises concern for compressive lesions, particularly posterior communicating artery aneurysms. In this case, thin-slice MRI excluded an aneurysm and mass lesions. Separately, CTA revealed atherosclerotic plaques in the cavernous segment of the internal carotid arteries; however, no direct nerve compression or hemodynamically significant stenosis was observed. These plaque findings were considered incidental and not the primary cause of the oculomotor nerve palsy. CSF examination showed no malignant cells or infectious markers. While microvascular ischemia less commonly presents with pupil involvement, it remains a consideration. However, the absence of vascular risk factors, subacute progression, and—most importantly—dramatic response to immunosuppressive therapy with normalization of serum IgG4 levels, collectively support an inflammatory mechanism related to IgG4-RD rather than ischemic or compressive etiology.

In addition to compressive effects, IgG4-RD can involve neural tissue through several interrelated mechanisms. A core immunologic disturbance is T helper 2 cell/regulatory T cell imbalance within affected tissues, where T helper 2 cell-derived IL-4 and IL-13 promote differentiation of B cells into IgG4 + plasma cells while simultaneously activating orbital fibroblasts via upregulation of pro-fibrotic factors such as platelet-derived growth factor-B and lysyl oxidase-like 2, fostering a self-sustaining cycle of inflammatory infiltration and fibrosis (23, 24). Follicular helper T (Tfh) cells contribute by producing interleukin-21 (IL-21), further enhancing B-cell antibody production. A multicenter study reported elevated circulating T cell immunoreceptor with Ig and ITIM domains Tfh cells in active IgG4-RD, with IL-21 production positively correlating with serum IgG4 levels and disease activity scores, providing a cellular basis for localized hypergammaglobulinemia (25). Autoantigens and antibodies may contribute to IgG4-RD pathogenesis. Anti-galectin-3 antibodies correlate positively with total IgG and IgG4 levels (*p* = 0.05 and *p* = 0.03, respectively), with markedly higher prevalence in lymph nodes than in normal tissue (26). Annexin A11 has been identified as a target recognized by both IgG4 and IgG1 autoantibodies; IgG1 anti-annexin A11 antibodies exhibit pro-inflammatory activity, whereas IgG4 antibodies may competitively inhibit this response by epitope binding, implicating annexin A11 as a potential driver of immune dysregulation in IgG4-RD (27). However, these autoantibodies have primarily been described in pancreatic, biliary, nodal, and salivary gland involvement, with no reported associations in neurologically manifesting IgG4-RD. In this case, given the absence of imaging abnormalities, we hypothesize that autoantibodies, inflammatory cytokines, and hypergammaglobulinemia may have contributed to oculomotor nerve injury. Cytokine profiling or autoantibody testing was not performed; if relapse occurs, a comprehensive immunologic evaluation may help elucidate the underlying mechanisms.

IgG4-related disease (IgG4-RD) exhibits clinical heterogeneity, yet treatment principles are largely consistent. Glucocorticoids remain first-line therapy, typically initiated at moderate-to-high doses (prednisone 0.6–1.0 mg/kg/day), with pulse therapy reserved for cases involving the nervous system (28). Dose–response analyses indicate that low-dose regimens (<0.39 mg/kg/day) are associated with higher relapse rates compared with medium-dose regimens (0.40–0.69 mg/kg/day), suggesting inadequate efficacy at lower doses; paradoxically, very high-dose regimens (>0.7 mg/kg/day) also show higher relapse rates than medium doses, likely reflecting greater baseline disease severity in patients requiring aggressive initial therapy. Consequently, initial dosing of prednisolone in the range of 0.40–0.69 mg/kg/day is considered appropriate (29). In our patient, the initial dose of 40 mg/day (approximately 0.6 mg/kg/day) was selected to balance efficacy and safety, given his advanced age.

Immunosuppressive agents serve as steroid-sparing adjuncts to reduce relapse risk and facilitate tapering of glucocorticoids. Commonly used agents include azathioprine, mycophenolate mofetil (MMF), leflunomide, methotrexate, and cyclophosphamide (29, 30). Rituximab has been incorporated into updated guidelines as a second-line therapy and may be considered third-line after inadequate response to glucocorticoids or failure of conventional immunomodulators (31). Clinical remission rates with rituximab range from 88.9 to 97%, with relapse rates of 21.9–23.3%, demonstrating superior efficacy compared with glucocorticoids alone or in combination with conventional immunosuppression (32, 33). Although rituximab is a standard treatment regimen, it was not chosen for this patient due to its high cost, the patient’s advanced age, and the elevated risk of infection. For refractory disease, tocilizumab (IL-6 receptor antagonist) or abatacept (T-cell co-stimulation inhibitor) may be considered, although evidence remains limited to case reports (34, 35). In this patient, sustained disease control was achieved with a combination of glucocorticoids and MMF, with stable status during follow-up. Our treatment strategy, while effective in this individual case, represents a non-standard, individualized approach. This case illustrates that treatment decisions in elderly patients with IgG4-RD may require deviation from standard protocols based on patient preferences, comorbidity burden, and access to costly biologic agents.

IgG4-RD presenting with oculomotor nerve palsy generally carries a favorable prognosis, but two important long-term risks require ongoing surveillance. First, relapse commonly occurs within 1–2 years after treatment, often involving previously affected organs. Surveillance typically includes serial measurements of serum IgG4, IgG4/IgG ratio, and imaging of involved sites every three months for the first two years, followed by semiannual assessments for the next three years (36). Second, malignant transformation risk is elevated in IgG4-RD, with lymphoma incidence approximately 70-fold higher than in the general population (7). In patients with unexplained fever, weight loss, or lymphadenopathy, prompt PET-CT and biopsy should be pursued. Although our patient achieved significant clinical improvement, the relatively short follow-up of four months does not permit definitive conclusions regarding long-term relapse risk or malignant progression.

This report has several limitations. First, cytokine profiling and autoantibody testing were not performed, limiting definitive characterization of immune-mediated pathways that may contribute to nerve-specific injury. Second, while visual evoked potentials (VEP) were performed and were normal, electrophysiological studies specifically targeting the oculomotor nerve (such as blink reflex studies or quantitative electromyography of extraocular muscles) were not available, which could have provided additional objective evidence of nerve dysfunction. Third, the short follow-up duration restricts assessment of long-term relapse and malignant transformation risks. Fourth, no nerve biopsy was performed to confirm direct neural infiltration. Fifth, as a single-case report, external validity requires validation in larger cohorts. Despite these limitations, the case underscores the value of multidisciplinary collaboration in managing IgG4-RD, enabling comprehensive evaluation and optimized therapeutic decision-making. It also emphasizes that IgG4-RD should be considered in the differential diagnosis of unexplained cranial neuropathies, even when radiographic abnormalities are absent. Future research should aim to identify serum biomarkers predictive of neurological involvement and to refine treatment strategies for this rare presentation.

From the patient’s perspective, the diagnostic delay was a source of substantial frustration. The initial evaluation in the hematology department, when neurological symptoms had already emerged, compounded his anxiety about a possible malignant cause. He reported that ptosis and diplopia significantly affected his daily activities, including reading and walking. Fortunately, after treatment and clinical improvement, he returned to his baseline functional status and expressed satisfaction with the outcomes. This case underscores that, beyond laboratory and imaging endpoints, the patient’s quality of life and emotional well-being are critical measures of successful management in IgG4-RD.

## Conclusion

IgG4-related disease should be considered in the differential diagnosis of unexplained cranial neuropathies. This case documents a rare instance of isolated oculomotor nerve palsy as the predominant manifestation of IgG4-RD and highlights that neurological recovery may be gradual, requiring sustained immunosuppression.

## Data Availability

The original contributions presented in the study are included in the article/Supplementary material, further inquiries can be directed to the corresponding author.

## Supplementary material

SUPPLEMENTARY FIGURE S1 Multimodal imaging findings. **(A–D)** ^18^F-FDG PET/CT images show increased tracer uptake in the mediastinal **(A)**, mediastinal and axillary **(B)**, and iliac vascular **(C)** lymph nodes, as well as the whole-body distribution of lesions **(D)**. **(E–G)** Head and neck CTA reveals severe stenosis at the origin of the left vertebral artery **(E)**, and calcified atherosclerotic plaques in the cavernous segment of the left **(F)** and right **(G)** internal carotid arteries. **(H–L)** Contrast-enhanced brain MRI shows the course and morphology of the oculomotor nerves. No obvious thickening or abnormal enhancement is observed in the left **(H–J)** or right **(K,L)** oculomotor nerve.

SUPPLEMENTARY FIGURE S2 Clinical photograph showing right ptosis and impaired ocular motility **(A)**. Post-treatment clinical image demonstrating restored oculomotor nerve function **(B)**.

SUPPLEMENTARY FIGURE S3 Visual evoked potential (VEP) findings. The waveforms and latency-amplitude parameters for both eyes were within normal reference ranges, indicating intact visual pathway function.
