# Supplementary figures and images for: Case Report: IgG4-related disease presenting with prominent oculomotor nerve palsy
Source: Front Neurol. 2026 Jul 9;17:1829911. doi: 10.3389/fneur.2026.1829911 (PMC13391401; doi:10.3389/fneur.2026.1829911)

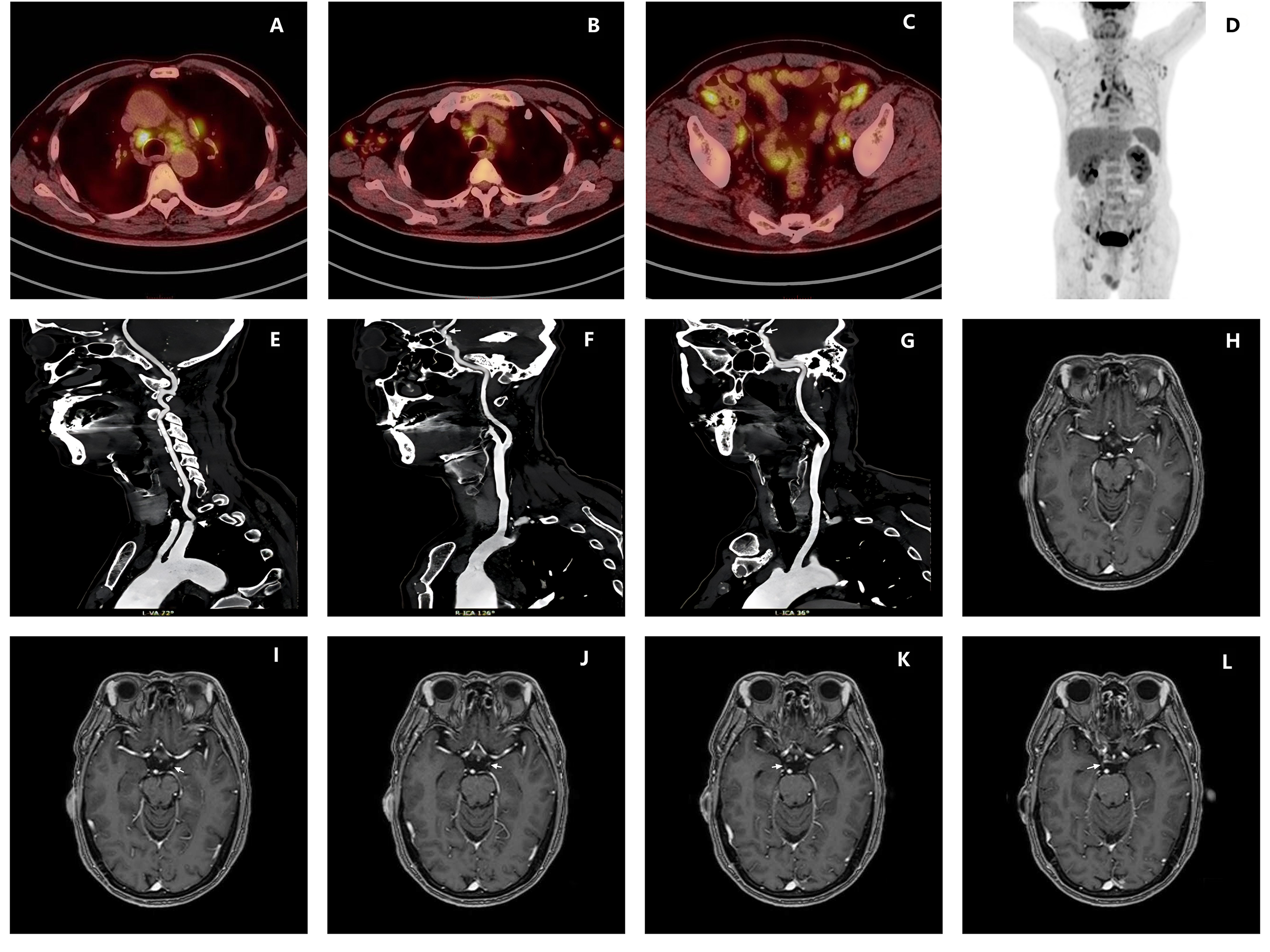

Supplement: SUPPLEMENTARY FIGURE S1 — Multimodal imaging findings. (A–D) 18F-FDG PET/CT images show increased tracer uptake in the mediastinal (A), mediastinal and axillary (B), and iliac vascular (C) lymph nodes, as well as the whole-body distribution of lesions (D). (E–G) Head and neck CTA reveals severe stenosis at the origin of the left vertebral artery (E), and calcified atherosclerotic plaques in the cavernous segment of the left (F) and right (G) internal carotid arteries. (H–L) Contrast-enhanced brain MRI shows the course and morphology of the oculomotor nerves. No obvious thickening or abnormal enhancement is observed in the left (H–J) or right (K,L) oculomotor nerve. [file Image_1.TIF]

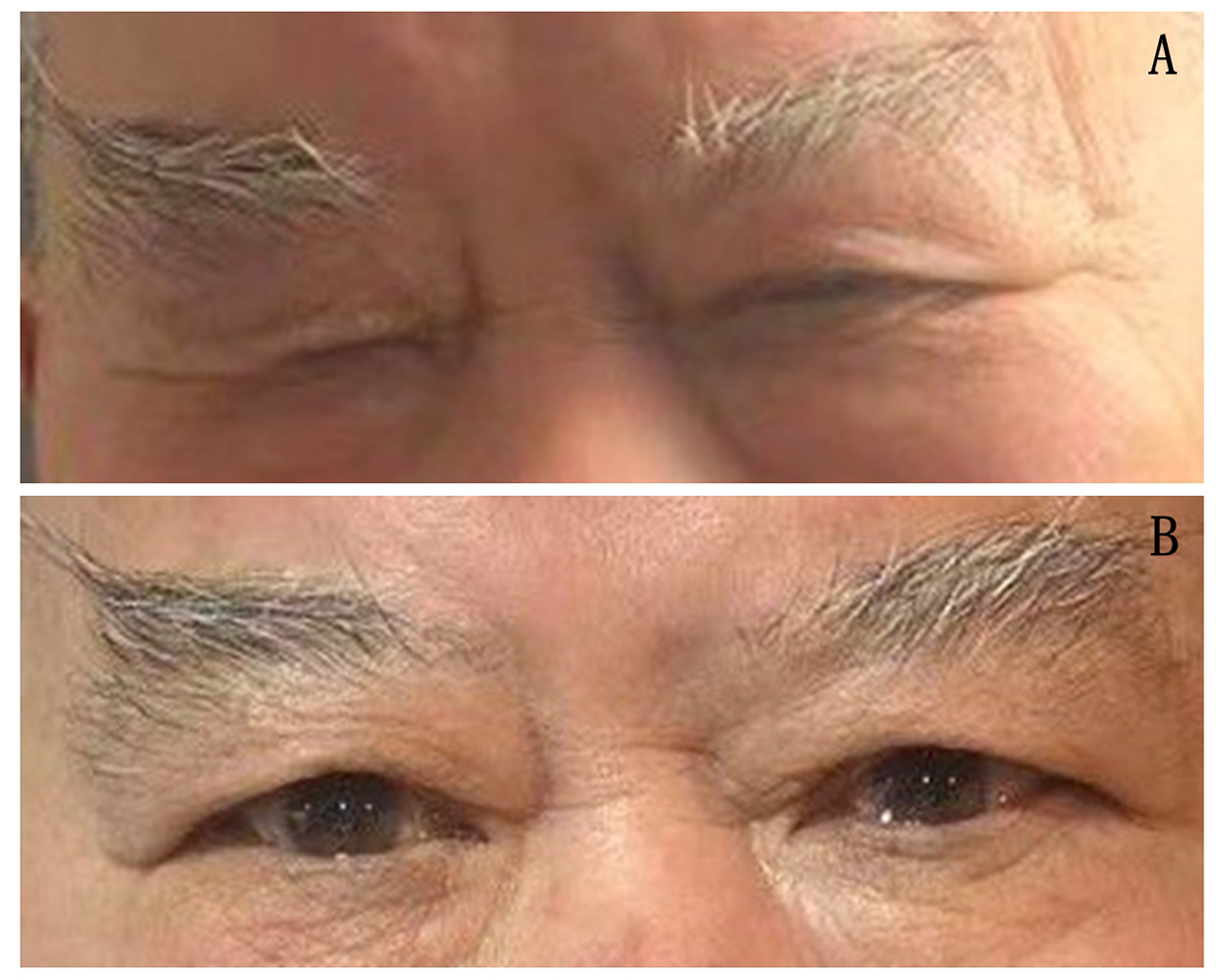

Supplement: SUPPLEMENTARY FIGURE S2 — Clinical photograph showing right ptosis and impaired ocular motility (A). Post-treatment clinical image demonstrating restored oculomotor nerve function (B). [file Image_2.TIF]

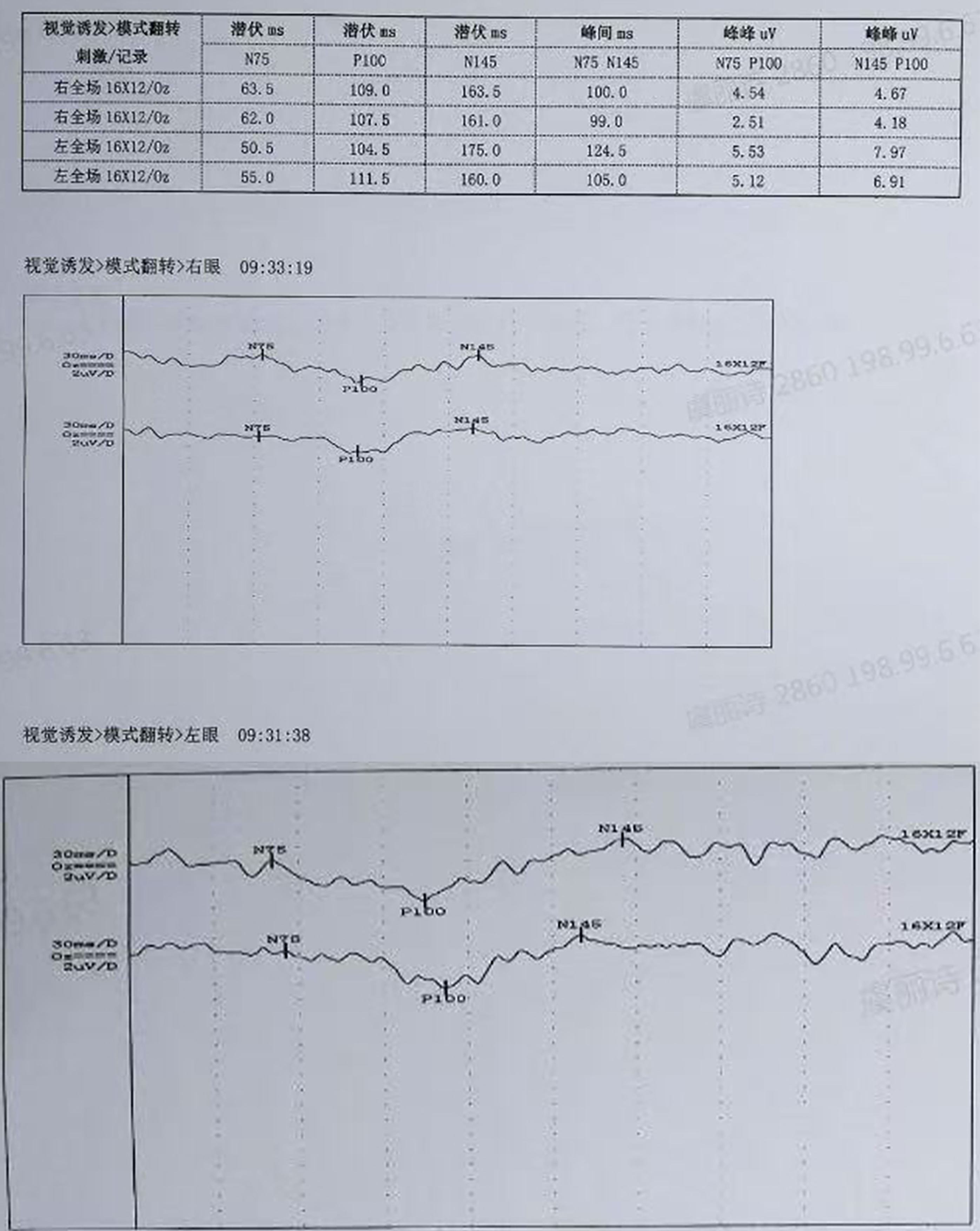

Supplement: SUPPLEMENTARY FIGURE S3 — Visual evoked potential (VEP) findings. The waveforms and latency-amplitude parameters for both eyes were within normal reference ranges, indicating intact visual pathway function. [file Image_3.TIF]
