# Supplementary material for: Case Report: IgG4-related disease presenting with prominent oculomotor nerve palsy
Source: Front Neurol. 2026 Jul 9;17:1829911. doi: 10.3389/fneur.2026.1829911 (PMC13391401; doi:10.3389/fneur.2026.1829911)
Supplement: Supplementary file 5 [file Table_1.DOCX]

**Supplementary table1. Cerebrospinal Fluid (CSF) Analysis Results**

| Item | Result | Reference Range |
| --- | --- | --- |
| Chloride（mmol/L） | 122.8 | 120-132 |
| Total Protein（g/L） | 0.63 | 0.2-0.4 |
| Glucose（mmol/L） | 3.82 | 2.5-4.5 |
| Clarity | Clear | Clear |
| White Blood Cells（/ul） | 2 | 0-8 |
| Red Blood Cells（/ul） | 0 | 0 |
| Pandy’s Test | Positive | Negative |
